# Exercise ameliorates insulin resistance and improves ASK1‐mediated insulin signalling in obese rats

**DOI:** 10.1111/jcmm.16994

**Published:** 2021-11-03

**Authors:** Yong Zhang, Tingting Ye, Puqing Zhou, Runjing Li, Zuofeng Liu, Jianyuan Xie, Tianmiao Hua, Qingyan Sun

**Affiliations:** ^1^ Physiology laboratory of College of Life Sciences Anhui Normal University Wuhu China; ^2^ The State Key Laboratory of Cellular Stress Biology Innovation Center for Cell Biology School of Life Sciences Xiamen University Xiamen China; ^3^ Department of Cardiology School of Medicine The First Affiliated Hospital of Xiamen University Xiamen University Xiamen China; ^4^ Department of Hepatobiliary Surgery School of Medicine Xiang'an Hospital of Xiamen University Xiamen University Xiamen China; ^5^ Neurobiology laboratory of College of Life Sciences Anhui Normal University Wuhu China

**Keywords:** ASK1, exercise, insulin resistance, insulin signalling transduction

## Abstract

Increasing evidence reveals that physical exercise is an efficient therapeutical approach in the treatment of insulin resistance (IR) and related metabolic diseases. However, the potential beneficial effects of exercise on insulin resistance and its underlying mechanisms remain unclear. Recent findings elucidated the negative role of ASK1 in repressing the glucose uptake through JNK1‐IRS1‐Akt signalling in liver. Thus, a detailed investigation of the effect of ASK1‐mediated insulin signalling on exercise‐mediated improvement of insulin sensitivity and its underlying mechanism was implemented in this study. Using a high‐fat diet‐induced IR rat model of chronic or acute swimming exercise training, we here showed that body weight and visceral fat mass were significantly reduced after chronic exercise. Moreover, chronic exercise reduced serum FFAs levels and hepatic triglyceride content. Both chronic and acute exercise promoted glucose tolerance and insulin sensitivity. Meanwhile, both chronic and acute exercise decreased ASK1 phosphorylation and improved JNK1‐IRS1‐Akt signalling. Furthermore, exercise training decreased CFLAR, CREG and TRAF1 protein levels in liver of obese rats, which are positive regulator of ASK1 activity. These results suggested that swimming exercise demonstrated to be an effective ameliorator of IR through the regulation of ASK1‐mediated insulin signalling and therefore, could present a prospective therapeutic mean towards the treatment of IR and several metabolic diseases based on IR, containing NAFLD and type Ⅱ diabetes.

## 1 INTRODUCTION

Insulin is an important metabolic hormone synthesized by pancreatic β cells, which stimulates peripheral tissues, such as skeletal muscle and adipose tissue, for glucose uptake. In addition, insulin also inhibits liver glucose output and adipose tissue lipolysis.^1^ Insulin resistance (IR) is defined as the inability of a known quantity of insulin to increase glucose uptake and utilization in target tissues and organs, as much as it does in a normal physiological status.^2^ Insulin resistance leads to an increase in blood glucose and circulating free fatty acids (FFAs), and ultimately to a range of pathologic conditions, including type 2 diabetes, cardiovascular disease and non‐alcoholic fatty liver disease.^3^

The liver regulates insulin sensitivity primarily by regulating glucose and lipid metabolism, which controls the body's energy needs. FFAs play a significant role in hepatic insulin resistance because increased plasma FFA levels inhibit insulin‐induced endogenous glucose production.^4^ With the development of insulin resistance, the flow of FFAs from adipose tissue to the liver increases, and during the excessive accumulation of fatty acids in the liver, the liver's ability to convert fatty acids to triglycerides (TG) increases, which in turn lead to impaired insulin signalling and aggravated hepatic and systemic insulin resistance.^5^, ^6^, ^7^

Apoptosis signal‐regulated kinase 1 (ASK1), a member of the mitogen‐activated protein kinase kinase (MAP3Ks) family, is an upstream activator of c‐Jun N‐terminal kinase 1 (JNK1) signalling cascades, and it plays a pivotal role in hepatic lipid metabolism and insulin resistance.^8^ Mechanistically, ASK1 is enabled by autophosphorylation at the T845 domain under pathological state, which serves as the primary activator of its downstream cascades. As downstream effector of ASK1, JNK1 directly facilitated obesity‐related insulin resistance and ectopic hepatic lipid accumulation by promoting serine phosphorylation of IRS1 and inhibiting of Akt signalling in hepatocytes.^8^, ^9^ In turn, activation of the ASK1‐JNK1 pathway can induce the expression of key gluconeogenesis related genes, it contains phosphoenolpyruvate carboxykinase (PEPCK) and glucose‐6‐phosphatase (G6Pase), which in turn inhibits the synthesis of liver glycogen.^10^, ^11^

Further researches revealed that there are several upstream regulators of ASK1 activity, which dictates its ultimate effects. TNF receptor‐associated factor 1 (TRAF1) expressed by hepatocytes has recently shown to play an important role in regulating obesity, insulin resistance and hepatic lipid accumulation, which is largely dependent on enhancing the activation of ASK1‐mediated JNK1‐IRS1‐Akt cascades.^12^ CASP8 and FADD‐like apoptosis regulator (CFLAR) directly interrupts the N‐terminus‐mediated dimerization of ASK1, and thus reducing its subsequent signal activation of JNK1 among the hepatocytes of subjects with obesity‐related insulin resistance.^13^ Dickkopf‐associated protein 3 (DKK3) is a regulator of the Wnt signal and is a negative upstream regulator of ASK1. Mechanistically, DKK3 interacted with ASK1 and thus inhibited the activation of the downstream JNK1‐mediated insulin signalling pathway.^14^ The cellular repressor of E1A‐stimulated genes (CREG) is another novel cellular target that affects ASK1 activity, which directly interacts with ASK1 and inhibits its phosphorylation, thereby blocking the downstream JNK1 signalling pathway and dramatically reducing obesity‐insulin resistance.^15^ Caspase Recruitment Domain Protein 6 (Card6) is a typical member of the CARD family, which is a novel negative regulator of IR that was confirmed in high‐fat diet (HFD)‐induced IR mice and in genetically obese mice. Card6 protects against IR by interacting with and suppressing activation of ASK1 and its subsequent JNK1 signalling.^16^

Several studies have demonstrated the role of exercise in improving insulin sensitivity and promoting glucose homeostasis.^17^, ^18^ However, these mechanisms are not fully understood. The molecular mechanisms of exercise enhancing insulin sensitivity may be related to the altered expression and/or activation of key proteins regulating glucose metabolism. In the present study, we investigated this hypothesis that ASK1‐mediated insulin signalling pathway may play an important role in liver ameliorating insulin resistance in obese rats and aimed at providing basic experimental evidence for clinical practice.

## 2 MATERIALS AND METHODS

### 2.1 Experimental animals and grouping

Forty 7‐week‐old male Sprague‐Dawley rats, weighing 250–270 g were purchased from Nanjing Qing‐Long‐Shan Animal Breeding Farm (Nanjing, China, Certificate: No. SX1207). All the rats were housed at a controlled temperature of 24–26°C and humidity of 50%–60% in a 12‐h light/dark cycle with free access to chow and sterile water. All experimental protocols were reviewed and authorized by the local animal ethics committee (Anhui Normal University, Wuhu, China). One week after feeding adaptation, the rats were randomly divided into normal control (NCT) group, high‐fat diet sedentary (SD‐HFD) group, high‐fat diet plus chronic exercise (CE‐HFD) group and high‐fat diet plus acute exercise (AE‐HFD) group (*n* = 10 per group). The NCT group was fed a normal diet (65% carbohydrates, 13% fat and 22% protein), while the other groups were all fed an HFD (40% carbohydrates, 38% fat and 22% protein) for 16 weeks.

### 2.2 Chronic exercise schemes

Weight‐loaded swimming training was performed in this study, gave that researches have shown that weight‐bearing exercise training can improve physical fitness.^19^, ^20^ The CE‐HFD group rats participating in the experimental chronic exercise intervention received swimming training with 5% load of body weight in a plastic cylindrical tank of 60 cm depth and 55 cm diameters, and the temperature of the water was maintained at 34 ± 1.5°C. Swimming training was conducted for 60 min once a day, five times a week, for 8 weeks (from 10 to 17th week). Forty‐eight hours after the final session of swimming exercise, the rats were anaesthetized by single‐dose intraperitoneal injection of 10% chloral hydrate.

### 2.3 Acute exercise schemes

The AE‐HFD group rats swam for two 3‐h periods with 45 min rest periods for a total swimming time of 6 h, under the identical conditions conducted as the chronic exercise training at 17th week. The NCT and SD‐HFD group rats remained in the same experimental condition; however, the depth of water used was up to the thorax level to minimize the water‐induced stress.^21^ The AE‐HFD group rats were euthanized with 10% chloral hydrate at sixteen hours after the last bout of exercise. Body weight of all the rats was monitored and recorded weekly during exercise training course (from 10 to 17th week).

### 2.4 Fasting glucose, oral glucose tolerance test (OGTT) and serum insulin determination

For the evaluation of glucose tolerance, all the rats were given glucose orally (2.0 g/kg BW) after overnight fasting prior that the animals were euthanized. Blood samples were collected from the tail vein at 0, 15, 30, 60, 90 and 120 min, and glucose was measured using a glucose metre (Roche, Germany). The serum was centrifuged at 4℃ (4000 rpm, 900 × *g*) for 15 min and stored at −80℃ for assay. Serum insulin level was determined by commercially available insulin ELISA kit (CUSABIO, Wuhan). The zero‐time value was taken as the fasting blood glucose value and fasting insulin value. The homeostatic model assessment of insulin resistance (HOMA‐IR) index was calculated according to the following formulas:

HOMA‐IR = (FBG × FINS)/22.5,

where FBG is the fasting blood glucose level and FINS is the fasting insulin level.

The insulin sensitivity index was calculated by the ratio of area under the glucose curve to area under the insulin curve (AUCg/AUCi).

### 2.5 Blood and tissues samples collection

In order to reduce the acute effects of exercise itself, CE‐HFD group rats were anaesthetized at 48 h. After the last swimming training, the group of AE‐HFD was fasted for 12 h, and then given 10% chloral hydrate intraperitoneally injected for anaesthesia at 16 h later. The blood was collected from the abdominal aorta by vacuum sampling, and the serum was separated and preserved as described above, which were used for detection of blood biochemical indicators. The visceral adipose tissue and liver were separated, rinsed and weighed, frozen in liquid nitrogen and stored at −80°C for later use.

### 2.6 Biochemical assays for serum and liver samples

Serum FFA levels were detected by FFA assay kit (Solarbio, China). The TG content in lipid extract from liver tissue were determined by TG kit (SolarBio, China). Liver glycogen assay kit (Solarbio, China) was used to determine the content of glycogen in liver fragments. All experimental procedures were carried out according to manufacturer's instructions.

### 2.7 Tissue extraction and Immunoblotting analysis

Liver tissue was lysed on ice with RIPA lysis buffer, and protease inhibitors, phosphatase inhibitors and 1mM PMSF (Beyotime, China) were added. The extracts were centrifuged at 12,000 rpm (8000 × *g*) and 4°C for 15 min to remove insoluble material, and the supernatants were used for protein quantification, performed by the BCA method. Equal amounts of proteins (30–90 μg) were separated on 8%–12% SDS‐PAGE gels, and then transferred onto PVDF membranes (Beyotime, China). Membranes were subsequently blocked with 5% skimmed milk. The membranes were incubated with following primary antibodies: anti‐TRAF1, anti‐CFLAR, anti‐DKK3, anti‐CREG anti‐Card6 and anti‐ASK1 from Proteintech (USA); anti‐phospho‐ASK1 (Thr845), anti‐SAPK/JNK, anti‐phospho‐SAPK/JNK (Thr183/Tyr185), anti‐IRS1, anti‐phospho‐IRS1 (Ser307) and anti‐Akt from Cell Signaling Technology (USA), anti‐phospho‐Akt (Ser473) from Abcam (USA); anti‐GAPDH from Sangon Biotech (China). After washing with 0.1% Tween 20 in TBS, the membranes were incubated with HRP‐conjugated secondary antibody (Sangon Biotech) and bound antibody was detected by BeyoECL Star (Beyotime, China). Bands were quantified and analysed by Image Pro Plus software.

### 2.8 Statistical analysis

Data were reported as mean ±SD for each group. The t test and one‐way analysis of variance were used for statistical comparison between groups. All statistical analyses were performed using GraphPad Prism 5.0 software, *p* < 0.05 is statistically significant.

## 3 RESULTS

### 3.1 Swimming training improves physiological and metabolic parameters in IR rats

**FIGURE 1:**
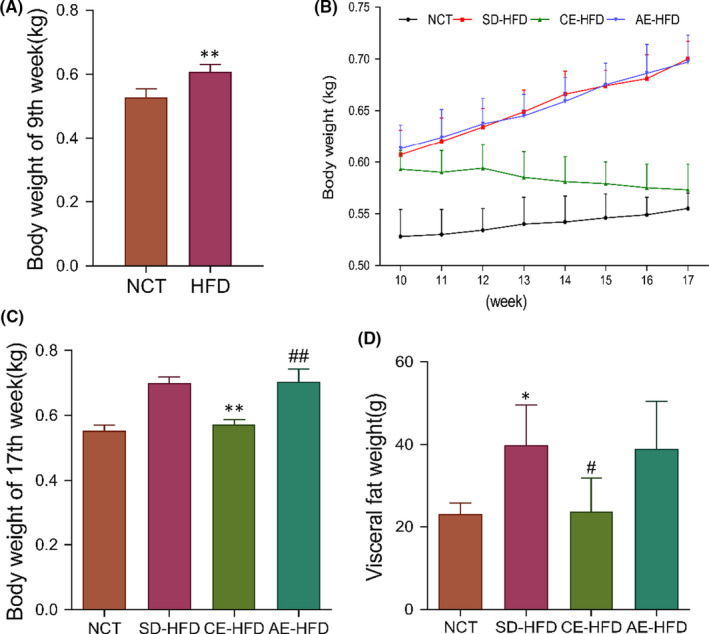
Physiology parameters in normal control rats, SD‐HFD rats and obese rats submitted to chronic and acute exercise procedure. (A) Body weight of 9th week, (B) Body weight during the exercise training period, (C) Body weight of 17th week, and (D) Visceral fat weight. Data are presented as mean ±SD. *n* = 6–10. *p < 0.05, **p < 0.01 versus NCT group; #*p* < 0.05, ##*p* < 0.01 versus SD‐HFD group. Abbreviations: AE‐HFD, high‐fat diet plus acute exercise; CE‐HFD, high‐fat diet plus chronic exercise; NCT, normal control; SD‐HFD, high‐fat diet sedentary

The body weight of experimental rats was measured during swim training for 8 weeks. Compared with the normal rats, the body weight (BW) and visceral fat mass (VFM) of obese rats increased significantly. Eight‐week swimming training markedly reduced the BW and VFM; however, acute swimming training had no significant alleviation on BW and VFM (Figure 1A–1D).

**FIGURE 2:**
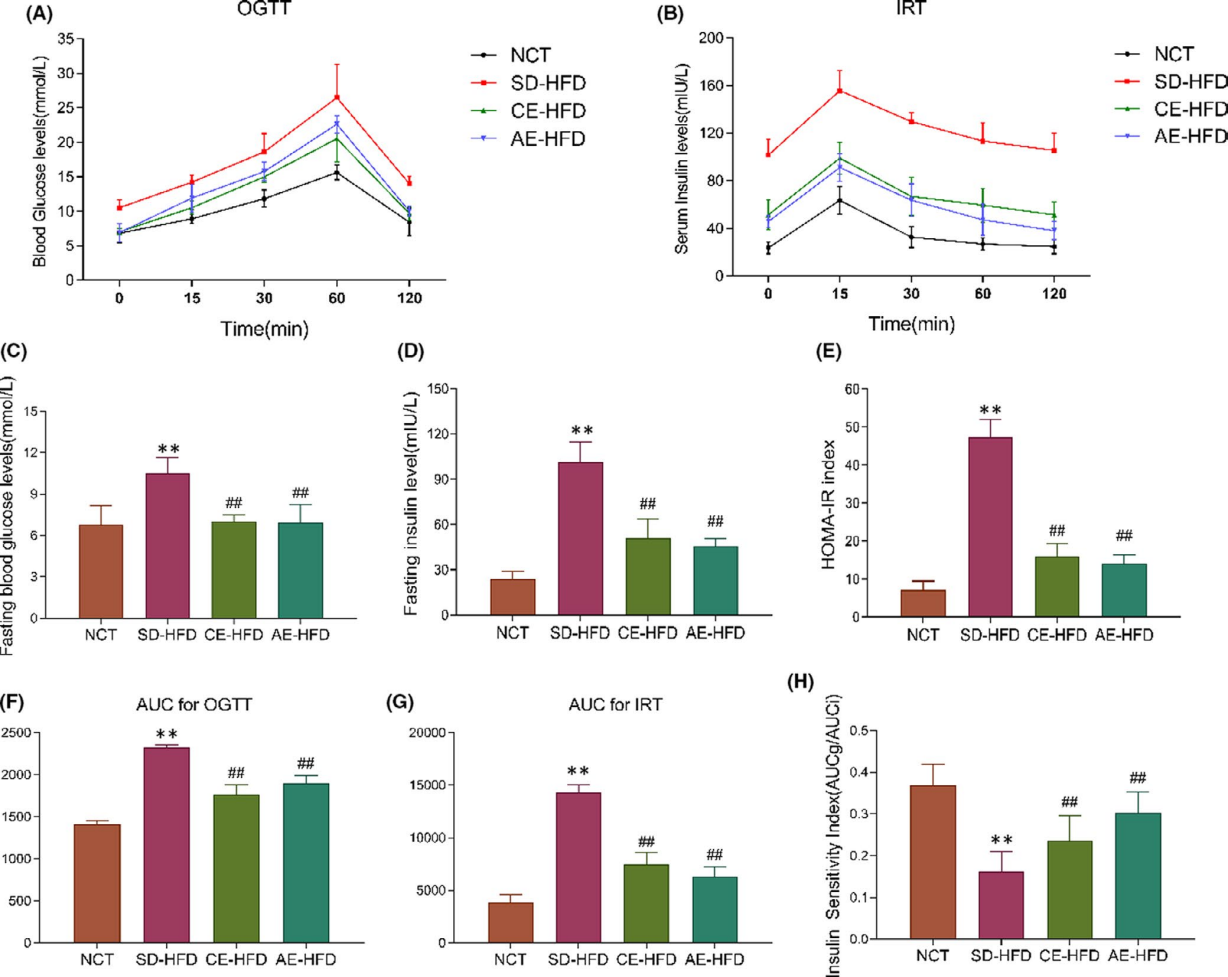
Metabolic parameters in normal control rats, SD‐HFD rats and obese rats submitted to chronic and acute exercise procedure. (A) Glucose homeostasis was assessed by an oral glucose tolerance test (OGTT), (B) Insulin release test (IRT), (C) Fasting blood glucose levels (FBG), (D) Fasting insulin levels (FINS), (E) HOMR‐IR index was calculated using (FBG ×FINS)/22.5, (F) area under the curve for OGTT (AUCg), (G) area under the curve for IRT (AUCi) and (H) insulin sensitivity index was calculated using the AUCg/AUCi. Data are presented as mean ±SD. *n* = 6–10. **p < 0.01 versus NCT group; ##*p* < 0.01 versus SD‐HFD group. Abbreviations: AE‐HFD, high‐fat diet plus acute exercise; CE‐HFD, high‐fat diet plus chronic exercise; IRT, Insulin release test; NCT, normal control; OGTT, oral glucose tolerance test; SD‐HFD, high‐fat diet sedentary

Glucose tolerance and insulin sensitivity were assessed by OGTT assay. The results demonstrated that the obese rats presented reduced glucose tolerance and insulin response when compared with that of the normal rats, and the CE‐HFD and AE‐HFD rats exhibited an enhance in glucose tolerance compared to SD‐HFD rats (Figure 2A, 2B). In addition, FBG and FINS were measured and HOMA‐IR was calculated for assessment of IR, AUC for OGTT and IRT were also calculated for evaluation of insulin sensitivity index (ISI). These results showed that FBG, FINS and HOMA‐IR were significantly increased, whereas ISI was significantly reduced in SD‐HFD group rats. In addition, compared with the SD‐HFD group, FBG, FINS and HOMA‐IR were significantly reduced, ISI was significantly increased inversely in CE‐HFD and AE‐HFD groups (Figure 2C–2H).

### 3.2 Effects of swimming training on serum FFAs, hepatic lipid accumulation and gluconeogenesis

**FIGURE 3:**
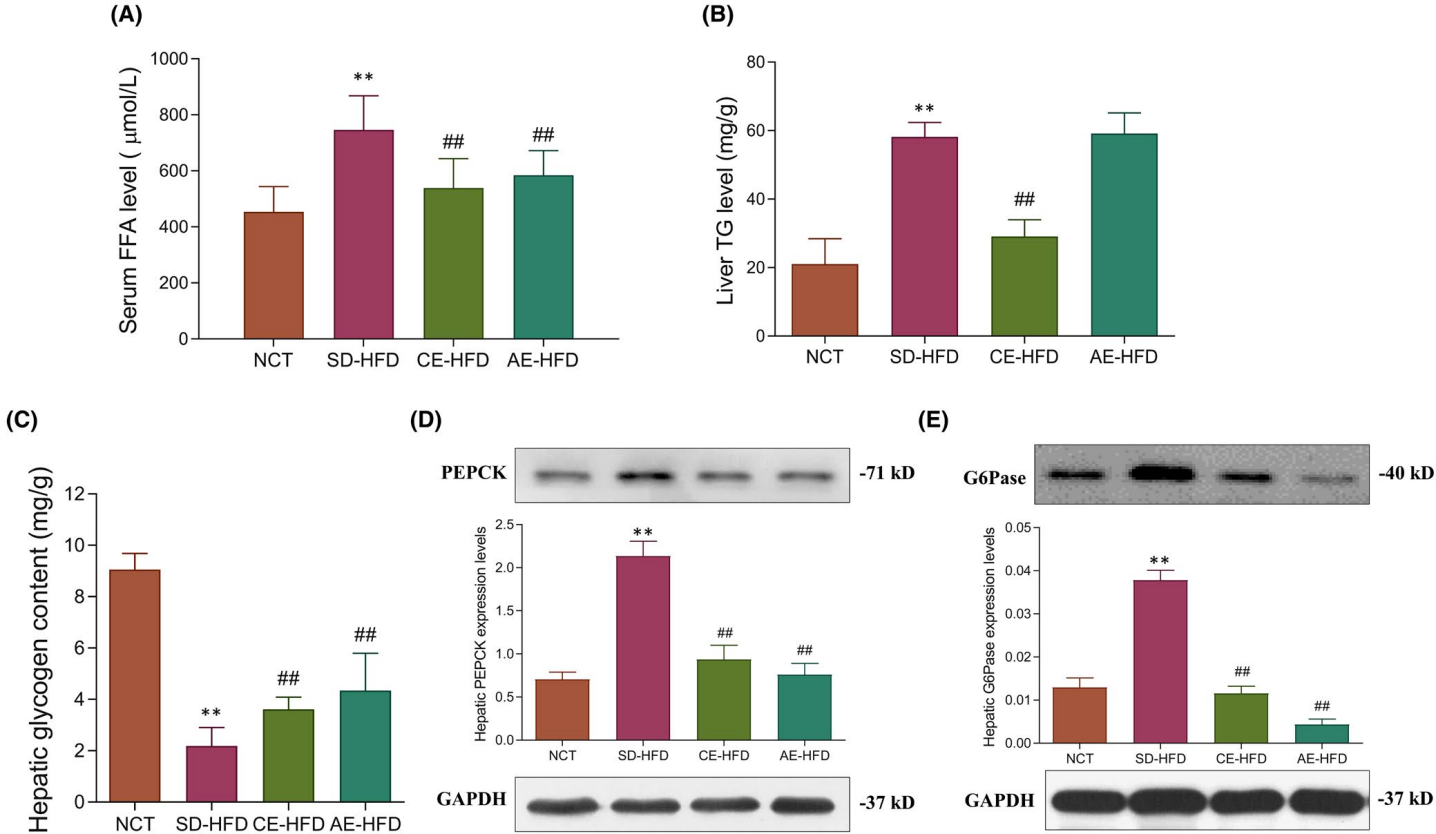
Effects of swimming training on serum FFAs, hepatic lipid accumulation and gluconeogenesis. (A) Serum FFA concentrations, (B) Liver TG content, (C) liver glycogen content, (D) Western blot analysis of the protein levels of PEPCK and (E) protein levels of G6Pase. GAPDH served as the loading control. Data are presented as mean ±SD. *n* = 6–10. All experiments were repeated at least 3 times independently. **p < 0.01 versus NCT group; ##*p* < 0.01 versus SD‐HFD group. Abbreviations: AE‐HFD, high‐fat diet plus acute exercise; CE‐HFD, high‐fat diet plus chronic exercise; NCT, normal control; SD‐HFD, high‐fat diet sedentary; TG, triglyceride

To further evaluate the effects of swimming training on lipid and glucose metabolism in IR rats, we detected serum FFAs, hepatic TG and glycogen contents. Compared with the NCT group rats, the levels of serum FFAs and hepatic TG contents were significantly elevated (Figure 3A, 3B). Furthermore, glycogen contents were reduced, while gluconeogenesis related key gene expression, PEPCK and G6Pase, were significantly increased in liver of SD‐HFD group rats (Figure 3C–3E). After 8‐week chronic swimming training, the CE‐HFD group rats presented reduced hepatic TG accumulation and serum FFAs levels, and the hepatic gluconeogenesis was weakened, demonstrated by increased glycogen contents, and decreased PEPCK and G6Pase protein levels in liver (Figure 3A–3E). Similarly, acute swimming training also reduced serum FFAs levels, and hepatic glycogen contents were increased. Meanwhile, hepatic gluconeogenesis was attenuated, accompanied by decreased PEPCK and G6Pase protein levels (Figure 3A–3E).

### 3.3 Swimming exercise training improved ASK1‐mediated insulin signalling pathway

**FIGURE 4:**
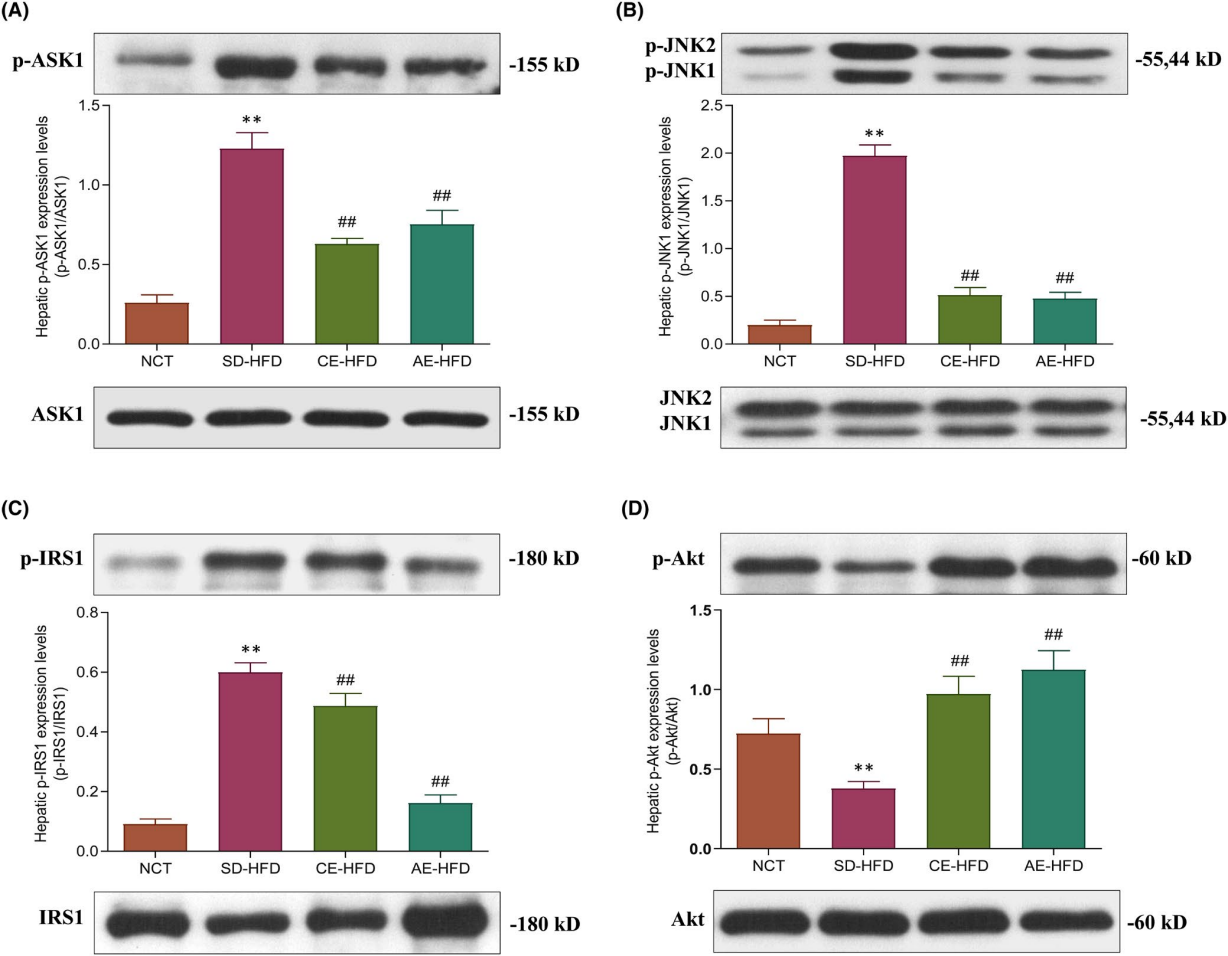
Expression analysis of proteins within the ASK1‐mediated signalling in liver of normal control rats, SD‐HFD rats and obese rats submitted to chronic and acute exercise procedure. (A) Western blot analysis of the protein level of phosphorylated ASK1‐Thr845, (B) phosphorylated JNK1‐Thr183/Tyr185, (C) phosphorylated IRS1‐Ser307 and (D) phosphorylated Akt‐Ser473. Data are presented as mean ±SD. All experiments were repeated at least 3 times independently. **p < 0.01 versus NCT group; ##*p* < 0.01 versus SD‐HFD group. Abbreviations: AE‐HFD, high‐fat diet plus acute exercise; CE‐HFD, high‐fat diet plus chronic exercise; NCT, normal control; SD‐HFD, high‐fat diet sedentary

Next, we evaluated the effects of swimming exercise training on those proteins involved in insulin signalling in the liver. Western blot analysis revealed high‐fat diet‐induced impairment of insulin signalling transduction in SD‐HFD group rats when compared with NCT group rats, as demonstrated by decreased phosphorylation of Akt at serine 473 and increased phosphorylation of JNK1 and IRS1. Following chronic and acute exercise training, the rats in CE‐HFD group and AE‐HFD group respectively presented improved insulin signalling transduction, as demonstrated by increased phosphorylation of Akt and decreased phosphorylation of JNK1 and IRS1 (Figure 4b–4d). Furthermore, ASK1 has been demonstrated to regulate insulin signalling via JNK1‐IRS1‐Akt signalling. Our results disclosed that ASK1 phosphorylation in liver was increased in SD‐HFD group compared with NCT group. Rats in CE‐HFD group and AE‐HFD group undergoing respective exercise training showed decreased ASK1 phosphorylation (Figure 4A). These findings indicated that exercise training could improve ASK1‐mediated insulin signalling in liver of HFD‐induced IR rats.

### 3.4 Effects of exercise training on regulators for TAK1 phosphorylation

**FIGURE 5:**
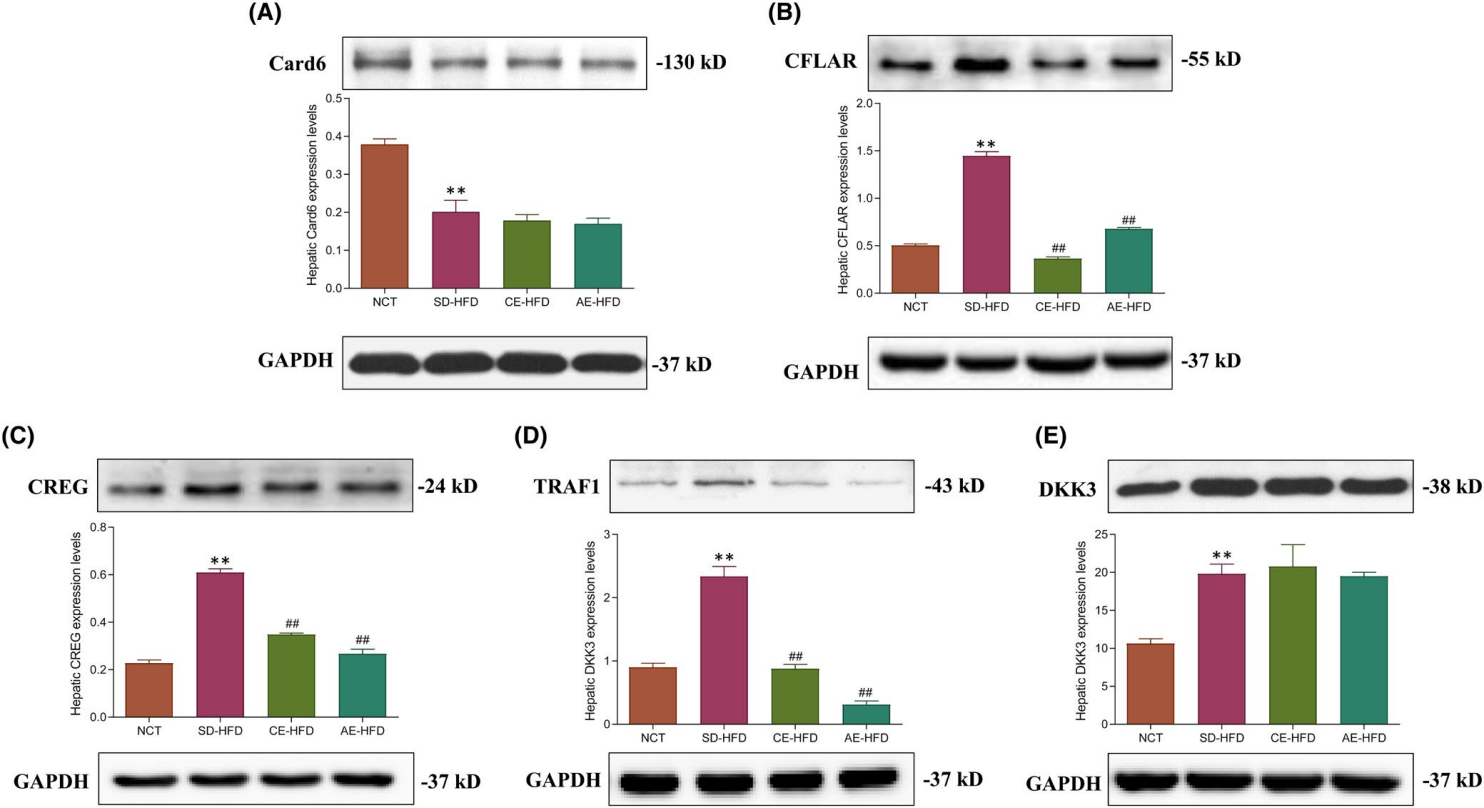
Proteins levels analysis of the regulators for ASK1 phosphorylation in liver of normal control rats, SD‐HFD rats and obese rats submitted to chronic and acute exercise procedure. (A) Western blot analysis of the protein level of Card6, (B) protein level of CFLAR, (C) protein level of CREG, (D) protein level of TRAF1 and (E) protein level of DKK3. GAPDH served as the loading control. Data are presented as mean ±SD. All experiments were repeated at least 3 times independently. **p < 0.01 versus NCT group; ##*p* < 0.01 versus SD‐HFD group. Abbreviations: AE‐HFD, high‐fat diet plus acute exercise; CE‐HFD, high‐fat diet plus chronic exercise; NCT, normal control; SD‐HFD, high‐fat diet sedentary

Card6 function as pivotal suppressor of ASK1 phosphorylation, while CFLAR, CREG, TRAF1 and DKK3, all function as the upstream positive regulators for ASK1 phosphorylation; hence, we determined their protein levels in liver via Western blot method. The results of Western blot analysis disclosed that Card6 protein levels in liver of SD‐HFD group rats were significantly decreased compared to NCT group rats. Following chronic and acute exercise training, the protein level had no significant changes (Figure 5A). Furthermore, the protein levels of CFLAR, CREG, TRAF1 and DKK3 were significantly up‐expressed in liver of SD‐HFD group rats when compared with NCT group rats. Following chronic and acute exercise training, CFLAR, CREG and TRAF1 protein levels appeared significant downregulation (Figure 5b–5d). However, both chronic and acute exercise training had no significant effect on DKK3 protein expression (Figure 5e).

## 4 DISCUSSION

Physical exercise, as efficient therapeutic approach, has been verified to be effective in alleviating body IR induced by HFD, and delaying relative metabolic diseases, such as diabetes, non‐alcohol fatty liver disease (NAFLD) and atherosclerosis. Regarding the mechanism behind this, exercise‐induced amelioration of insulin signalling transduction is widely explored and is deemed to act a pivotal role in the metabolic protection effects. However, the effects of exercise training on ASK1‐mediated insulin signalling in liver under IR status has not been intensively investigated.

In the present study, we evaluated the effects of exercise training on IR and made an inquiry for the potential molecular mechanism in obese rats. We mainly assessed IR status and relative physiological and metabolic parameters in rats. Sixteen‐week HFD feeding resulted in significant increase in BW and VFM in rats, and the SD‐HFD group rats presented obvious IR state, as demonstrated by significantly higher FBG, FINS and HOMA‐IR index. Further OGTT and IRT assays were more confirmed the SD‐HFD group rats possessed abnormal glucose metabolism. Furthermore, decreased hepatic glycogen content, and increased serum FFAs and hepatic TG, all these disclosed glycolipids abnormal metabolism is an important pathological event in the progression of IR.^17^, ^22^, ^23^

ASK1 plays a pivotal role in hepatic lipid metabolism and IR, which has been demonstrated to regulate insulin signalling via JNK1‐IRS1‐Akt signalling. Further researches revealed that there are several upstream regulators of ASK1 activity, including Card6, CFLAR, CREG, TRAF1 and DKK3, which dictated its ultimate effects on JNK1‐IRS1‐Akt signalling. The current research disclosed that HFD feeding enhanced ASK1 phosphorylation, JNK1 and IRS1 phosphorylation, while decreased Akt phosphorylation. In addition, gluconeogenesis related proteins were prominently up‐expressed, which were in line with preceding pathological mechanism researches in mice.^12^, ^13^, ^14^, ^15^, ^16^

To furtherly understand the specific mechanism of exercise prevention of IR, we detected ASK1‐mediated insulin signalling in the livers of all experimental groups. As showing in current study, the ASK1 phosphorylation, along with its downstream JNK1 and IRS1 phosphorylation were decreased, inversely the Akt phosphorylation was enhanced in CE‐HFD group. And serum FFAs level and liver TG content were decreased compared to SD‐HFD group. Further results disclosed reduced expression of CFLAR, CREG and TRAF1 following CE in obese rats, which were the upstream regulatory factor of ASK1 activity. The liver glycogen content increased significantly after CE, accompanied by the downregulation of PEPCK and G6Pase. All of these changes are associated with a notable detente in the status of IR. The results of this study suggested that long‐term exercise training could modulate upstream regulators of ASK1 activity and then targeting ASK1‐mediated signal transduction. Hence, the alteration in ASK1‐mediated signalling induced by CE is notable and may be considered to be the cause of the improvement in IR after CE.

A large number of studies have shown that moderate weight loss can enhance the insulin sensitivity of tissues and organs, accordingly improve IR.^17^, ^24^, ^25^ The results of this study showed significant weight loss in CE‐HFD rats after 8 weeks of exercise training, raising the question of whether the improvement in IR was a result of exercise per se or was simply a result of weight loss. To clarify this question, single‐use acute exercise training was established, which had no significant change in BW and VFW. The results indicated that acute exercise decreased protein levels of CFLAR, CREG and TRAF1, accordingly reduced phosphorylation of ASK1 and downstream JNK1 and IRS1, and enhanced phosphorylation of Akt at Ser473, as well as causing serum FFAs reduction, and increasing liver glycogen content, which is related to suppressed activation of gluconeogenesis‐associated kinases, demonstrated by reduced PEPCK and G6Pase protein contents in AE‐HFD group. And in terms of current data, the AE‐HFD group rats had further improved insulin sensitivity index compared to CE‐HFD group, however, whether this is a ss phenomenon or an experimental randomness, which can be further compared and verified in future studies.

Overall, our results show that both chronic and acute exercise training produced significant reductions in CFLAR, CREG and TRAF1, and ameliorates ASK1‐mediated insulin signalling. And two types of exercise training improved physiological and metabolic parameters in IR rats. Previous studies focused more on the variation of classical insulin receptor signalling pathways, while the intracellular signalling pathway regulated by ASK1 that we focused on is independent of insulin receptor. These results provided important advances in understanding the underlying molecular mechanisms that link exercise training to improved IR.

## CONFLICTS OF INTEREST

The authors declare that there are no conflicts of interest.

## AUTHOR CONTRIBUTIONS

**Yong Zhang:** Investigation (lead); Resources (lead); Writing‐original draft (lead); Writing‐review & editing (lead). **Tingting Ye:** Data curation (equal). **Puqing Zhou:** Investigation (equal). **Runjing Li:** Formal analysis (equal). **Zuofeng Liu:** Data curation (equal). **Jianyuan Xie:** Formal analysis (equal). **Tianmiao Hua:** Funding acquisition (equal). **Qingyan Sun:** Project administration (lead); Resources (lead); Writing‐original draft (lead).

## Data Availability

The data that support the findings are available from the corresponding author upon reasonable request.
